# The Endocannabinoid Anandamide Attenuates Acute Respiratory Distress Syndrome by Downregulating miRNA that Target Inflammatory Pathways

**DOI:** 10.3389/fphar.2021.644281

**Published:** 2021-04-27

**Authors:** Muthanna Sultan, Hasan Alghetaa, Amirah Mohammed, Osama A. Abdulla, Paul J. Wisniewski, Narendra Singh, Prakash Nagarkatti, Mitzi Nagarkatti

**Affiliations:** Department of Pathology, Microbiology and Immunology, School of Medicine, University of SC, Columbia, SC, United States

**Keywords:** acute respiratory distress syndrome (ARDS), anandamide (AEA), staphylococcus enterotoxin B (SEB), micro-RNA (miRNA/miR), myeloid derived suppressor cells (MDSC), T regulatory cells (T regs), miRNA-23a-3p, miRNA-34a-5p

## Abstract

Acute respiratory distress syndrome (ARDS) is defined as a type of respiratory failure that is caused by a variety of insults such as pneumonia, sepsis, trauma and certain viral infections. In this study, we investigated the effect of an endocannabinoid, anandamide (AEA), on ARDS induced in the mouse by *Staphylococcus* Enterotoxin B (SEB). Administration of a single intranasal dose of SEB in mice and treated with exogenous AEA at a dose of 40 mg/kg body weight led to the amelioration of ARDS in mice. Clinically, plethysmography results indicated that there was an improvement in lung function after AEA treatment accompanied by a decrease of inflammatory cell infiltrate. There was also a significant decrease in pro-inflammatory cytokines IL-2, TNF-α, and IFN-γ, and immune cells including CD4^+^ T cells, CD8^+^ T cells, Vβ8^+^ T cells, and NK^+^ T cells in the lungs. Concurrently, an increase in anti-inflammatory phenotypes such as CD11b + Gr1+ Myeloid-derived Suppressor Cells (MDSCs), CD4 + FOXP3 + Tregs, and CD4^+^IL10 + cells was observed in the lungs. Microarray data showed that AEA treatment in ARDS mice significantly altered numerous miRNA including downregulation of miRNA-23a-3p, which caused an upregulation of arginase (ARG1), which encodes for arginase, a marker for MDSCs, as well as TGF-β2, which induces Tregs. AEA also caused down-regulation of miRNA-34a-5p which led to induction of FoxP3, a master regulator of Tregs. Transfection of T cells using miRNA-23a-3p or miRNA-34a-5p mimics and inhibitors confirmed that these miRNAs targeted ARG1, TGFβ2 and FoxP3. In conclusion, the data obtained from this study suggests that endocannabinoids such as AEA can attenuate ARDS induced by SEB by suppressing inflammation through down-regulation of key miRNA that regulate immunosuppressive pathways involving the induction of MDSCs and Tregs.

## Introduction

Acute respiratory distress syndrome (ARDS), a pulmonary disease characterized by an exudation of protein-rich fluid into alveolar space (Zhao et al., 2017), has a high morbidity rate approaching 200,000 cases each year with an approximate mortality rate of 27–45% depending on disease severity (Diamond et al., 2020). ARDS is defined as a type of respiratory failure which is caused by a variety of insults such as pneumonia, sepsis, trauma, and certain viral infections. ARDS is usually accompanied by systemic hyperactivation of the immune system leading to inflammation in the lungs, development of pulmonary edema, alveolar damage, and often respiratory failure (Fan et al., 2018). As there are no pharmacological agents currently approved by the FDA to treat ARDS, there is a high rate of mortality associated with this disease.

Recently, the severe form of COVID-19 caused by infection with the SARS-CoV-2 virus has also been shown to elicit ARDS (Li and Ma, 2020), although the nature of pathogenesis may be different from other forms of ARDS. Nevertheless, ARDS is also accompanied by the release of inflammatory cytokines such as IL-6. IFN-γ and TNFα (Matthay and Zimmerman, 2005; Mohammed et al., 2020a; Mohammed et al., 2020b). In this context, suppressing pro-inflammatory responses is critical for attenuating disease severity.

The endocannabinoid system consists of the endocannabinoids, their metabolic enzymes and the cannabinoid receptors (Di Marzo, 2018). The analgesic effects of these endocannabinoids are primarily mediated through the cannabinoid receptor 1 (CB1) which has been shown to have a significant role in the presynaptic modulation of pain (Walker and Huang, 2002). In contrast, CB2 is primarily expressed on immune cells, and activation of CB2 has been shown to suppress inflammation (Ameri, 1999; Pandey et al., 2009), However, it should be noted that CB1 is also expressed on immune cells and its activation by cannabinoids can also induce immunosuppression (Sido et al., 2015b). Two of the best-characterized endocannabinoids are anandamide (AEA) and 2-arachidonoylglycerol (2-AG) (Devane et al., 1992; Mechoulam et al., 1995; Fan et al., 2018), which can bind to both CB1 and CB2 receptors (Barrie and Manolios, 2017). The main enzymes responsible for the hydrolysis of AEA and 2-AG are Fatty acid amide hydrolase (FAAH) and Monoacylglycerol lipase (MAGL), respectively (van Egmond et al., 2021) This has enabled the synthesis of many distinct classes of FAAH and MAGL inhibitors that have been shown to increase the levels of endocannabinoids when administered *in vivo*, thereby offering an opportunity to treat certain clinical disorders (van Egmond et al., 2021). Such FAAH and MAGL inhibitors have also been shown to effectively attenuate lipopolysaccharide (LPS)-induced acute lung inflammation (Costola-de-Souza et al., 2013; Wu et al., 2019; Abohalaka et al., 2020).

As recently reviewed, both AEA and 2-AG work in tandem as “master regulators” of the innate-adaptive immune axis, governing numerous immune responses (Chiurchiù et al., 2015). For example, we have shown that AEA attenuates type-IV delayed-type hypersensitivity (DTH) mediated by Th17 cells *via* the induction of IL-10 and miRNA (Jackson et al., 2014b) and that activated T and B cells produce 2-AG, thereby inhibiting T-cell activation and proliferation, and thus attenuating DTH (Sido et al., 2016). Taken together, the immunoregulatory effects of endocannabinoids are manifold, and may yet provide a suitable pharmacological intervention in the treatment of ARDS.

*Staphylococcus* Enterotoxin B (SEB) is a superantigen produced by the Gram-positive bacterium, *Staphylococcus aureus* that causes many diseases ranging from food poisoning to toxic shock syndrome. SEB is known to stimulate T cells *via* binding to the Major Histocompatibility Complex II (MHC II) outside the conventional antigen-binding site that shares the variable region of the T cell receptor causing a robust proliferation of T cells (Pinchuk et al., 2010). Because SEB is easily aerosolized, it is considered as a bioterrorism agent, and the Centers for Disease Control and Prevention (CDC) has classified SEB as Category B (Pinchuk et al., 2010).

Micro-RNAs (miRNAs) are highly conserved small non-coding RNA molecules (21–25 nucleotides) that are expressed in most organisms from plants to vertebrates and regulate gene expression by degrading or silencing their targeted mRNA (Pertwee and Waterhouse, 2005; Macfarlane and Murphy, 2010). Specifically, miRNAs use their seed sequence to interact with the 3′ untranslated region (3′UTR) found in the mRNA target *via* imperfect matching (Cannell et al., 2008). While many parameters govern miRNA-mRNA interactions, what adds to the inherent complexity between these interactions are the numerous bindings sites per miRNA and the potential of each mRNA to be targeted by multiple miRNAs (Krol et al., 2010; O’Neill et al., 2011). Consequently, miRNAs are involved in the regulation of several cellular processes including the regulation of immunity, particularly as it relates to innate immune responses in the process of pathogen clearance and tissue restitution (O’Neill et al., 2011). There is only one previous report, that from our laboratory, investigating whether the anti-inflammatory action of anandamide is associated with miRNA (Jackson et al., 2014). Additionally, we have also reported that the use of FAAH inhibitor enhances AEA *in vivo* and attenuates colitis through induction of miRNA that downregulates inflammatory pathways (Shamran et al., 2017).

Previous studies from our laboratory demonstrated that Delta-9-Tetrahydrocannabinol (THC), the principal psychoactive constituent of cannabis, can suppress SEB-mediated ARDS in mice (Mohammed et al., 2020b; Mohammed et al., 2020c). THC-treated mice showed significant alterations in the expression of miRNA in the lung-infiltrated mononuclear cells (MNCs), which were associated with suppression of lung inflammation (Mohammed et al., 2020a) Together, these studies suggested that cannabinoids have significant potential in the treatment of ARDS. Nonetheless, whether direct administration of endocannabinoids such as AEA can suppress ARDS has not been previously investigated. Such studies are essential because while THC and AEA are both know to activate CB1 and CB2 receptors, AEA has also been shown to act as an agonist for the Transient Receptor Potential Cation Channel Subfamily V member 1 (TRPV1), also known as the vanilloid receptor 1, THC does not modulate TRPV1 (Muller et al., 2018). In the current study, we, therefore, tested if endocannabinoid administration can suppress inflammation seen in ARDS caused by SEB. We found that AEA was highly effective in attenuating ARDS and inflammation in the lungs caused by SEB. We found that AEA altered the expression of miRNA in the mononuclear cells (MNC) isolated from the lungs of SEB-treated mice which promoted anti-inflammatory pathways. This study opens the possibility of the use of AEA or FAAH inhibitors in the attenuation of ARDS in a clinical setting.

## Materials and Methods

### Mice

Female C57BL/6 mice (6–8 weeks) were purchased from Jackson laboratories. All mice were housed under pathogen-free conditions in the Animal Resource Facility (ARF) at the University of South Carolina with a maximum of five animals per cage. All requirements for the experiments were performed under the policy approved by the Institutional Animal Care and Use Committee (IACUC). Mice were housed under a 12 h light/dark cycle at 18–23°C and 40–60% humidity.

### Chemicals, Reagents and Kits

All chemicals and reagents were purchased as follows: N-arachidonoyl ethanolamine or Anandamide (AEA) from Cayman Chemicals (Ann Arbor, Michigan, United States); *Staphylococcus* Enterotoxin B (SEB): from Toxin Technologies (Sarasota, FL); RPMI 1640 supported with L-glutamine: from Corning (New York, NY, United States); Fetal Bovine Serum, Penicillin and Streptomycin: from Invitrogen Life Technologies (CA, United States). True Nuclear Transcription Factor Buffer Set was purchased from Biolegend (San Diego, CA) while Fc Block reagent was purchased from BD Pharmingen (San Diego, CA). EasySep mouse MDSCs (CD11bGr1) selection kit was purchased from STEMCELL technologies (Seattle, WA) for purification of MDSCs. miScript SYBR green PCR kit, miScript primer assays kit, RNA easy, and miRNA easy kit were purchased from Qiagen (Valencia, CA). Taq DNA polymerase kit was purchased from Invitrogen Life Technologies (Carlsbad, CA).

### Induction of Acute Respiratory Distress Syndrome in Mice Using Staphylococcus Enterotoxin B (SEB) Inhalation and Treatment With Anandamide

ARDS was induced in mice as described previously (Rieder et al., 2011). Mice were randomly divided into either vehicle or treatment groups before exposure to SEB. Mice were exposed to SEB intra-nasally (I.N.) with a single dose at a concentration of 50 µg/mouse in 25 µl of Phosphate Buffer Saline (PBS) as previously described (Rieder et al., 2011) on day 0. On day-1, AEA or VEH was given into these mice by the Intra Peritoneal (I.P.) route at a dose of 40 mg/kg body weight. AEA dissolved in ethanol (50 mg/ml) was diluted further in PBS. Each mouse received 0.1 ml consisting of 84 μl of PBS and 16 μl of ethanol containing AEA. The vehicle controls received 0.1 ml consisting of 84 μl of PBS and 16 μl of ethanol without AEA. The dose of AEA was based on our previous studies demonstrating that 40 mg/kg body weight of AEA attenuated T cell-mediated delayed-type hypersensitivity response (Jackson et al., 2014b). The treatment with AEA was repeated on day 0 (SEB exposure day), and day 1. Mice were euthanized on day 2 (48 h after SEB exposure) for various studies.

### Evaluation of Lung Function

To evaluate the effect of AEA to attenuate the effects of SEB, clinical parameters of the lungs were measured using whole-body plethysmography (Buxco, Troy, NY, United States), as described (Elliott et al., 2016). A single mouse from each group of Naïve, SEB + VEH, and SEB + AEA was first restrained in a plethysmographic tube and was allowed to acclimatize as previously described (Elliott et al., 2016). The lung function was calculated for clinical parameters such as Specific Airway Resistance (sRAW), Specific Airway Conductance (sGAW) and Minute per Volume (MV).

### Lung Histopathology

Lung tissue was excised from each mouse and directly fixed in 10% formalin without inflation. The lung tissues were then embedded in paraffin and sections were cut at our core facility. The sections were then processed for H & E staining. Briefly, lung tissue sections mounted on slides were first transferred in xylene to deparaffinized the tissue sections. The tissue sections were then rehydrated in alcohol (100, 95, and 90%). The sections were finally stained with Hematoxylin and Eosin (H & E) and dehydrated. H & E stained sections were analyzed using KEYENCE digital microscope VHX-7000 (IL-United States).

### Isolation and Purification of Lung Mononuclear Cells (MNCs)

These cells were isolated as described previously (Rieder et al., 2012). Briefly, mice were given heparinized PBS to perfuse the lungs. A stomacher 80 biomaster blender (Seward, Fl) was used to homogenize the lung tissue suspended in 10 ml of 1× PBS and 10% Fetal Bovine Serum (FBS). The homogenized lung tissues were centrifuged at 1,200 rpm at 4°C for 12 min. The pellet was then washed with PBS and resuspended in PBS post centrifugation. We then used a density gradient centrifugation method to purify lung mononuclear cells (MNCs). In brief, the cells resuspended in sterile PBS were carefully layered using Histopaque-1077 purchased from Sigma-Aldrich (St. Louis, MO) and centrifuged at 500 × g for 30 min at room temperature (25°C). The mononuclear cells were collected at the interface. MNCs mixed with trypan blue were then enumerated using a Biorad TC 20-Automated cell counter.

### Detection of Cytokines in Serum

Blood samples from each group of mice were collected under general anesthesia. The collected blood samples were then centrifuged to collect the sera. The sera were either used immediately or stored at −80°C. The detection of the cytokines was performed using ELISA MAX standards kits from Biolegend.

### Flow Cytometry Analysis

Spleen cells and MNCs isolated from the lungs were stained with various fluorescent–conjugated antibodies purchased from Biolegend (SanDiego, CA, United States), which included: For T cell subsets: Allophycocyanin (APC) conjugated anti-CD3, Phycoerythrin (PE) conjugated anti-CD4, PerCP/Cyanine 5.5 (PerCP-CY5.5) conjugated anti-CD8, Fluorescein Isothiocyanate (FITC) conjugated anti-Vβ8, and PE/Dazzle Conjugated anti-NK1.1. For lymphocyte activation markers, T cell memory markers, MDSCs, and Tregs, the following Abs were used: Phycoerythrin (PE) Conjugated anti-CD69, Brilliant Violet-510 Conjugated anti-CD25, Fluorescein Isothiocyanate (FITC) Conjugated anti-CD44, Brilliant Violet-785 Conjugated anti-CD3, Allophycocyanin (APC) Conjugated anti-CD62L, Alexa Fluor 488 Conjugated anti-FoxP3, Phycoerythrin (PE) Conjugated anti-IL10, Fluorescein Isothiocyanate (FITC) Conjugated anti-CD11b, Phycoerythrin (PE) Conjugated anti-Gr1, Alexa Fluor 700 conjugated anti-LY6C, and Phycoerythrin cy5 (PECY5) conjugated anti-LY6G.

The staining of cells for dual markers was performed as described in our previous publications (Al-Ghezi et al., 2019; Becker et al., 2020; Dopkins et al., 2020; Mohammed et al., 2020b). In brief, MNCs and spleen cells were treated with Fc Block reagent (BD Pharmingen, San Diego, CA) followed by staining cells with various antibodies by incubation at 4°C for 20–30 min. Stained cells were washed twice with cold PBS containing 2% FBS (Staining buffer). For IL 10 and FOXP3 staining, we used the Intracellular Cytokine Staining Kit (BD biosciences) consisting of fixation/permeabilization buffer. In brief, the cells were fixed using fixation/permeabilization buffer, followed by washing the cells with PBS and then staining the cells using anti–Foxp3 and anti–IL10 antibodies. The cells were finally suspended in 0.5 ml staining buffer and analyzed using Beckman Coulter FC500 or BD Bioscience FACSCelesta^™^, which was followed by analysis on the FlowJo V10 version software.

### Induction of Myeloid-Derived Suppressor Cells by Anandamide

Mice were injected with Anandamide and SEB, as described earlier. Forty eight hrs later, mice were euthanized and the peritoneal exudate was collected and washed with PBS. The cells were resuspended in 1 ml, treated with Fc block for 10 min at RT. Next, the EasySep CD11b Gr1 selection kit (STEMCELL Technologies, Seattle, WA; catalog number 19867) was used to isolate CD11bGr1 based on the guidelines provided in the kit.

### Suppression of T Cell Proliferation by Myeloid-Derived Suppressor Cells *In Vitro*

To test the immunosuppressive effects of MDSCs on T cell proliferation, naive C57BL/6 mice were euthanized and splenic T cells were isolated. Splenic T cells at a concentration of 5 × 10^5^ cells/well were cultured in 96-well tissue culture plates in the presence of Concanavalin A (ConA), a T cell mitogen, at a concentration of (2.5 μg/ml) together with different ratios of AEA induced purified MDSCs for 48 h. (^3^H)thymidine (2 μCi per well) was added to the cell culture in the last 18 h, and the radioactivity was measured using a liquid-scintillation counter (MicroBeta TriLux; PerkinElmer).

### Micro-RNAs Arrays and Analysis

Micro-RNA analysis was performed as described previously (Tomar et al., 2015; Alghetaa et al., 2018). Total RNA including miRNA was isolated from lung infiltrating MNCs using miRNeasy kit from Qiagen. A Nanodrop 2000 spectrophotometer (Thermo Fisher Scientific, Wilmington, DE) was used to determine RNA purity and concentration. Next, Affymetrix gene chip miRNA 4.0 array platform was used (Affymetrix Inc., Santa Clara, CA) to determine the miRNA expression profile of lung MNCs and only those miRNAs that were altered more than 1.5-fold or higher were considered for further analysis. Ingenuity Pathway Analysis (IPA) was used for identifying the role of miRNA in various biological pathways using the software available at http://www.ingenuity.com. Also, the microRNA.org database was used to examine the sequence alignment regions between miRNA-23a-3p and miRNA-34a-5p and their respective target genes.

### Quantitative-Real Time Polymerase Chain Reaction (qRT-PCR)

This was performed as described previously (Mohammed et al., 2020a). Briefly, to determine the expression of the selected miRNA and respective genes, qRT-PCR was performed using cDNA synthesized from total RNA and miRNA. miScript II RT kit from Qiagen was used to synthesize cDNA using and following the protocol of Qiagen company.

Snord96A and GAPDH were used as endogenous controls. 2^ΔΔCt^ was used to determine fold change in expression the following primers were used for qRT-PCR (Table 1).

**TABLE 1:** The details of Primer sequences used for RT-qPCR.

Gene	Primer	Sequence
GAPDH	Forward	5-AGG​TCG​GTG​TGA​ACG​GAT​TTG-3
	Reverse	5-TGT​AGA​CCA​TGT​AGT​TGA​GGT​CA-3
FOXP3	Forward	5-CCC​ATC​CCC​AGG​AGT​CTT​G-3
	Reverse	5-ACC​ATG​ACT​AGG​GGC​ACT​GTA-3
TGFβ2	Forward	5-CTT​CGA​CGT​GAC​AGA​GGC​T-3
	Reverse	5-GCA​GGG​GCA​GTG​TAA​ACT​TAT​T-3
ARG1	Forward	5-CGG​GTT​AAA​TTC​GGG​TTA​TC-3
	Reverse	5-CGA​ACT​ACC​GCG​ATT​CTA​ATC-3

### Transfection of Micro-RNA

Transfection was performed as described previously (Busbee et al., 2015). In brief, splenocytes were cultured in complete RPMI 1640 medium (10% FBS, 10 mM L-glutamine, 10 mM Hepes, 50 *μ*M *β*-mercaptoethanol, and 100 *μ*g/ml penicillin). 2.5 × 10^5^ cells/well were seeded in a 24-well plate and transfected with mock control, synthetic mimic mmu-miRNA-23a-3p MSY0000532, synthetic mimic mmu-mmiRNA-34a-5p MIN0000532 using a HiPerFect transfection reagent kit from Qiagen.

### Statistics

Graph Pad Prism 6.0 Software (San Diego, CA, United States) was used for statistical analyses. Student’s *t*-test was used for comparison among two groups. One way-ANOVA with post hoc Tukey’s test was used to compare between more than two groups. The results were expressed as mean ± SEM. A *p*-value ≤ of 0.05 was considered statistically significant.

## Results

### Anandamide Improves Lung Function, Rescues Lung Damage, and Decreases Pro-Inflammatory Cytokines in Mice Exposed to Staphylococcus Enterotoxin B

**FIGURE 1:**
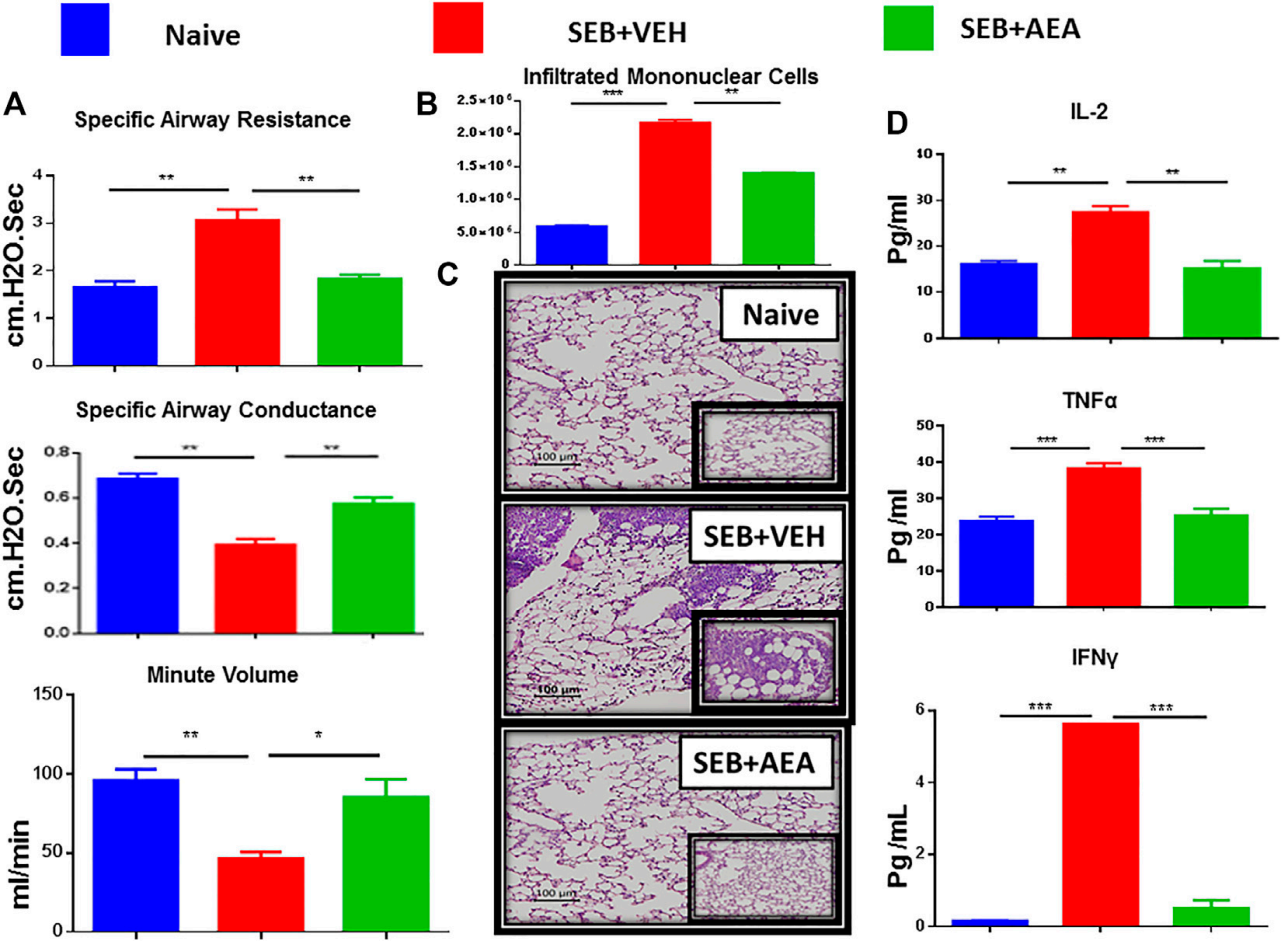
AEA attenuates SEB-induced ALI in mice. Mice were exposed to SEB intra-nasally with a single dose of 50 µg/mouse on day 0. On days-1, 0 and 1, AEA or VEH was given into these mice i. p at a dose of 40 mg/kg body weight. Mice were euthanized on day 2 for various studies. **(A)** Showing clinical functions of the lung including Specific Airways Resistance (sRAW), Specific Airways Conductance (sGAW), and Minute per Volume (MV). **(B)** Comparison between the groups for the total number of Mononuclear Cells isolated from the lungs. **(C)** Representative images of histopathological H & E staining of excised lung tissue (20X magnification). **(D)** Measurement of cytokines IL2, IL6, and TNFα in the serum. Five mice in each group were used and the data confirmed in three independent experiments. *p ≤ 0. 05, **p ≤ 0. 01, ***p ≤ 0. 001.

We used the following groups of mice to study the effect of AEA on SEB-mediated ARDS: Naïve, SEB + VEH, and SEB + AEA. After 48 h of SEB exposure, the lung function of all groups was evaluated using a Buxco instrument system. We observed that the clinical parameters for lung function including specific airways resistance, specific airway conductance, and minute per volume to be significantly improved in SEB + AEA groups and similar to the naïve group, in comparison to SEB + VEH mice (Figure 1A). These data demonstrated that AEA was able to rescue impairments to the lung function caused by SEB exposure. Further, the total number of mononuclear cells (MNCs) in the lung was significantly decreased in SEB + AEA mice when compared to SEB + VEH mice (Figure 1B). Histological sections of the lungs are shown in Figure 1C that are representative pictures of the data presented in Figure 1B.

Analysis of sera obtained from mice showed that there was a significant decrease in the pro-inflammatory cytokines TNFα, IL-2, and IFN-γ in SEB + AEA group when compared to SEB + VEH mice (Figure 1D).

### Anandamide Suppresses the Infiltration of Inflammatory Cells in the Lungs and Spleens

**FIGURE 2:**
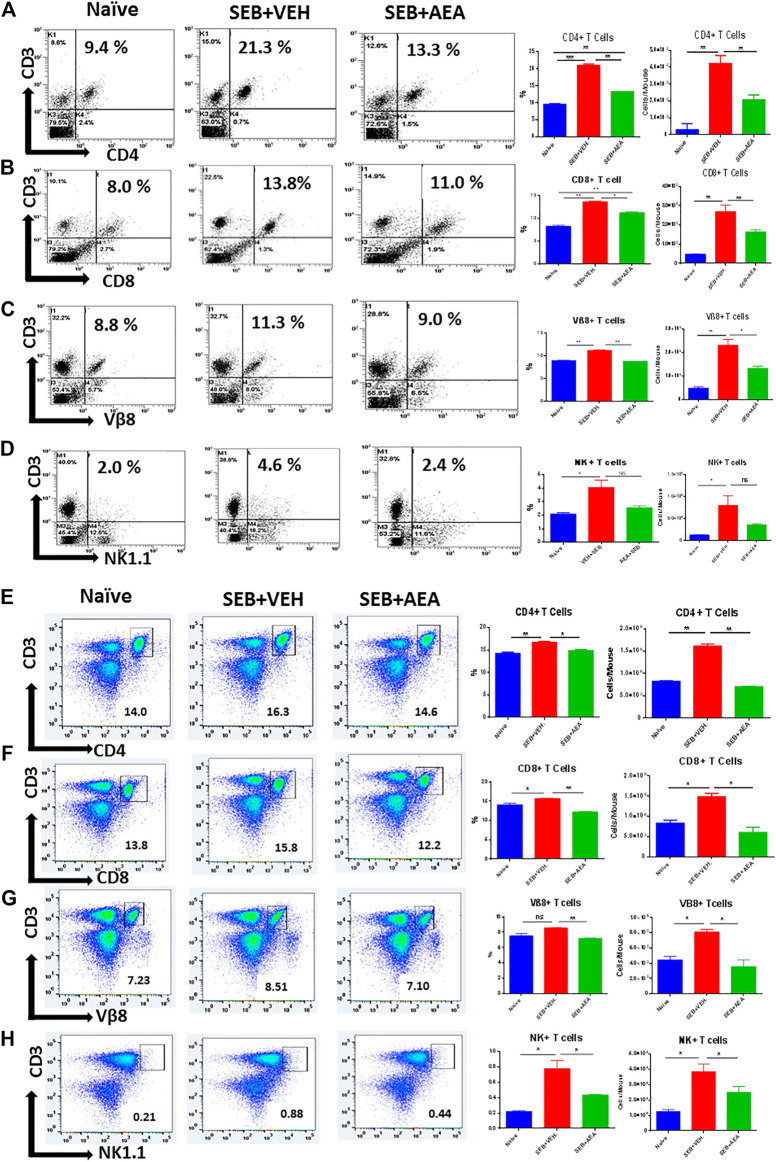
AEA decreases T cell subpopulations in the lungs and spleens. Mice were treated with SEB and AEA as described in Figure 1 legend. Each panel shows a representative experiment depicting lung MNCs and splenocytes analyzed for various T cell markers. Data from five mice/group is presented in the form of vertical bars with Mean+/-SEM. Panels A–D and E-H show data from the lungs and spleens, respectively. **(A,E)** CD3^+^CD4^+^ T cells **(B,F)** CD3^+^CD8^+^ T cells, **(C,G)** CD3+Vβ8+ T cells, **(D,H)** CD3+NK1.1 + cells. Five mice in each group were used and the data was confirmed in three independent experiments. *p ≤ 0. 05, **p ≤ 0. 01, ***p ≤ 0. 001.

We then assessed the presence of T cells in the lungs (Figures 2A–D) and spleens (Figures 2E–H) by staining with fluorescein-conjugated antibodies and analyzing using flow cytometry. The data have been depicted as a representative flow cytometric analysis followed by percentage of cells/mouse, and total number of cells/mouse in vertical bars. Results showed that there was a significant increase in T cell subset populations, both in percentages and numbers, including CD4^+^ T cells, CD8^+^ T cells, Vβ8+ T cells, and NK T cells in the lungs and spleens of SEB + VEH group when compared to the naïve mice, thereby demonstrating that SEB caused activation and proliferation of these cells (Figures 2A–H). Interestingly, SEB **+** AEA group when compared to the SEB + VEH group, showed a significant decrease in the percentage and numbers of all T cell subsets, including the Vβ8+ T cells that are specifically activated by SEB. This combined with the fact that SEB + AEA group had a decrease in the total number of infiltrating MNCs in the lungs (Figure 1B) when compared to SEB + VEH group, clearly demonstrated that AEA decreases both the percentage and total numbers of T cell subsets induced by SEB.

### Anandamide Suppresses the Proliferation of Splenic Lymphocytes *In Vitro*

**FIGURE 3:**
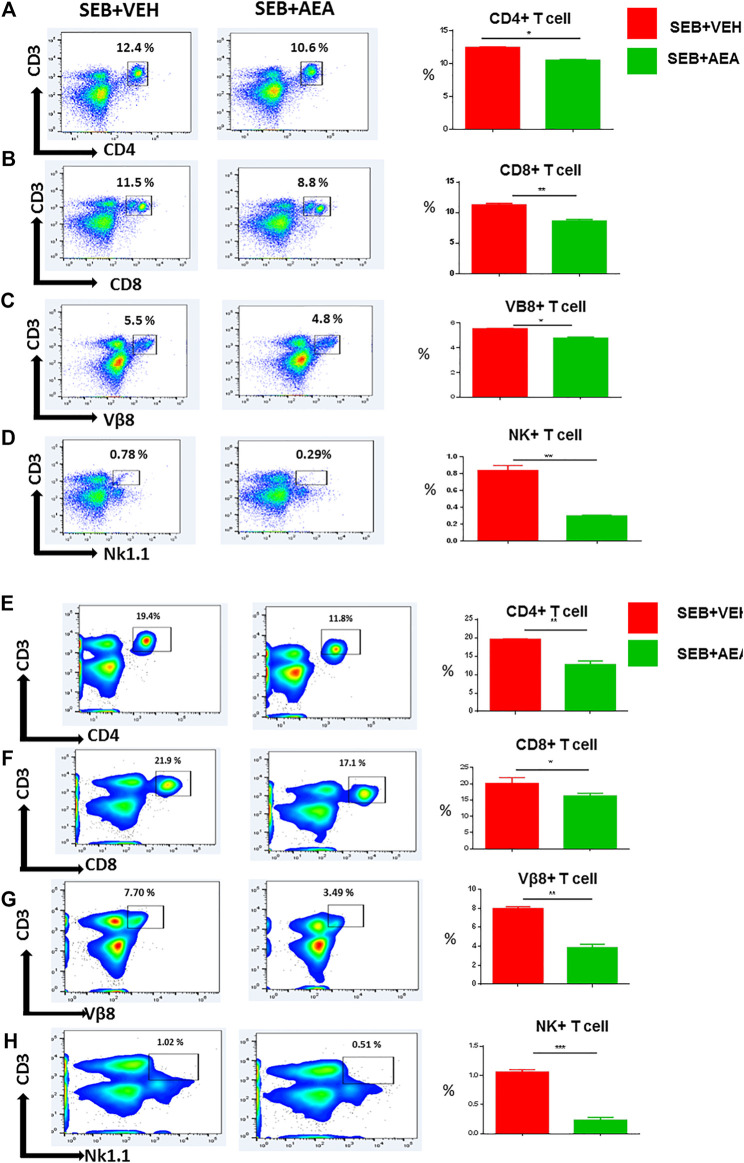
AEA decreases T cell subsets in the splenocytes activated with SEB *in vitro*. Spleen cells isolated from naïve mice were pretreated with AEA *in vitro* followed by activation with SEB, and then cultured for 24 **(A-D)** or 48 **(E-H)** hrs, and stained for various markers. Each panel shows a representative experiment using flow cytometry and the vertical bars depict data from groups of five mice with Mean+/-SEM. **(A,E)** CD3^+^CD4^+^ cells. **(B, F)** CD3^+^CD8^+^ cells. **(C,G)** CD3+Vβ8+ cells **(D,H)** CD3+NK + cells. The data was confirmed in three independent experiments. *p ≤ 0. 05, **p ≤ 0. 01.

**FIGURE 4:**
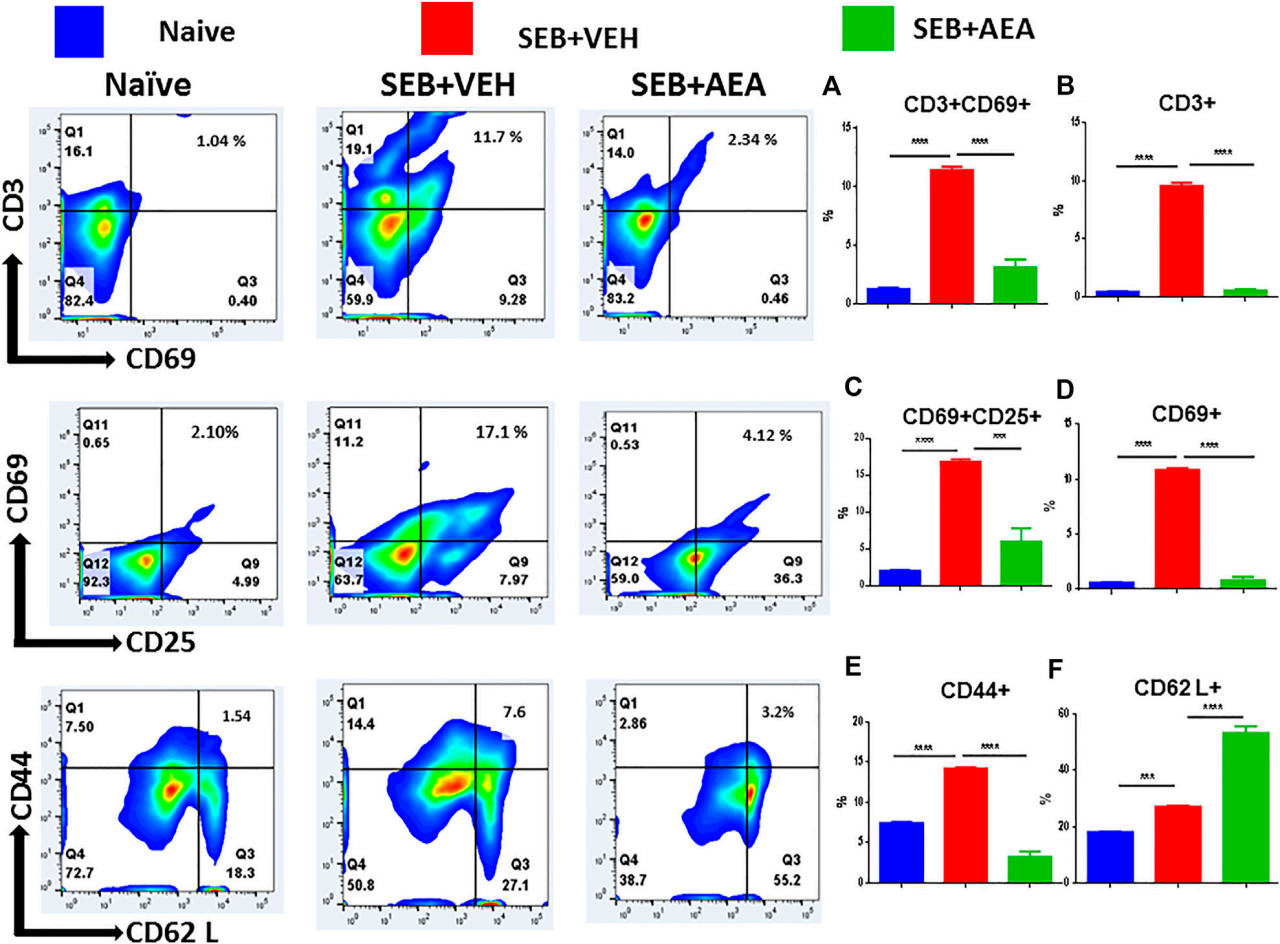
AEA suppresses T cell activation markers in splenocytes activated with SEB. The spleen cells were pretreated with AEA and then activated with SEB *in vitro* for 48 h as described in Figure 3 legend. The cells were stained for various activation markers. Each panel shows a representative experiment using flow cytometry, and the vertical bars depict percentage data from groups of five mice with Mean+/-SEM. **(A)** CD3^+^CD69 ^+^ cells, **(B)** CD3^+^ cells, **(C)** CD69 + 25+ cells, **(D)** CD69 ^+^ cells, **(E)** CD44 ^+^ cells, **(F)** CD62L + cells. *p ≤ 0. 05, **p ≤ 0. 01, ***p ≤ 0. 001.

To determine whether the AEA suppresses SEB-activated proliferation of T cells directly, we performed *in vitro* assays in which splenic lymphocytes, isolated from naïve mice, were pretreated with a dose of 20 μM of AEA and activated 30 min later with 1 μg/ml of SEB. The cells were then incubated for 24 and 48 h. We assessed the effect of AEA on T cell subpopulations, including CD4^+^ T cells, CD8^+^ T cells, Vβ8 + T cells, and NKT cells by flow cytometry. We observed that there was a significant decrease in the percentage of all T cell subsets and NKT cells in the SEB + AEA group when compared to the SEB + VEH group at 24 h (Figures 3A–D), and this decrease was further augmented at 48 h (Figures 3E–H). Next, we investigated activation markers such as CD3, CD69, CD25, and CD44, as well as CD62L (L-slectin) which is expressed on naïve CD4^+^ T cells used for recirculation and binding to high endothelial venules and on central memory T cells. We observed that the percentages of CD3^+^CD69^+^, CD69^+^, CD44^+^, and CD62L + cells from the SEB + Veh group were significantly increased when compared to naïve mice indicating that SEB had activated a significant proportion of T and other immune cells. However, in the SEB + AEA group, there was a significant decrease in percentages of these cells when compared to the SEB + VEH group except CD62L + cells which were enhanced in SEB + AEA mice when compared to the SEB + VEH group (Figure 4). It should be noted that when we looked at CD25 ^+^ cells that were CD69-, these cells were significantly increased SEB + AEA group (33.6%) when compared to SEB + VEH (7.9%) (Figure 4). Because CD25 is also expressed on Tregs, these data suggested that AEA may increase the proportion of Tregs which is shown in subsequent Figs. Together, these *in vitro* studies demonstrated that AEA directly worked on SEB-activated immune cells thereby significantly suppressing their activation.

### Anandamide Alters the Expression of Micro-RNAs in Lung Infiltrating Mononuclear Cells

**FIGURE 5:**
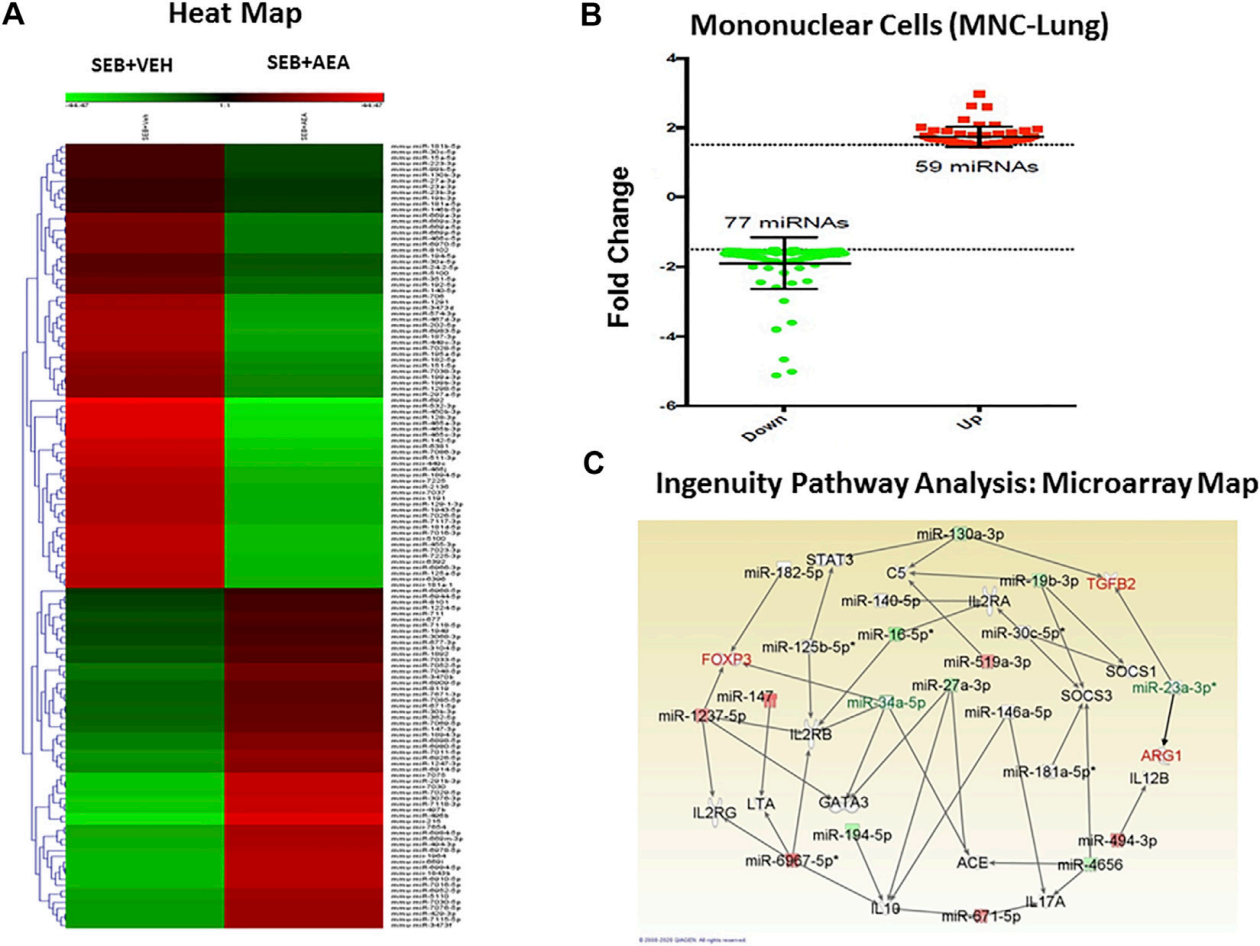
AEA alters miRNA expression in the mononuclear cells isolated from the lungs of SEB injected mice. Mice were treated with SEB and AEA as described in Figure 1 legend. Isolated mononuclear cells of the lung were screened for miRNA expression as described in methods **(A)**. The heat map that shows all of the altered and unchanged miRNAs in the VEH + SEB and AEA + SEB groups. **(B)** Diagram of miRNAs with 1.5 fold change showing 59 miRNAs upregulated while 77 miRNAs downregulated. **(C)** Networking of the miRNAs with their targeted genes including anti-inflammatory genes, the map showing the main miRNAs including miRNA 23a-3p targeting ARG1 and TGFβ2 gene while miRNA 34a-5p targeting FOXP3 gene.

We next analyzed miRNA profiles of lung infiltrating MNCs to examine the extent to which AEA attenuates inflammation *via* miRNA induction. The data were analyzed using Ingenuity Pathway Analysis (IPA) software as described earlier (Rao et al., 2015a). The heat map of miRNAs (>3,000) was first generated which showed marked alterations in miRNA expression in SEB + VEH mice when compared to the SEB + AEA mice. Upon analysis of a ≥1.5 fold increase or decrease in miRNA expression, a marked difference in the upregulation/downregulation of miRNAs between SEB + VEH and SEB + AEA mice was observed (Figure 5A). Specifically, there were at least 77 miRNAs that were upregulated and 59 miRNAs that were downregulated in SEB + AEA mice when compared to SEB + VEH mice (Figure 5B). Next, we analyzed select miRNAs for their targets especially those involved in the regulation of inflammation, using the IPA software from Qiagen and observed that some of the downregulated miRNAs, specifically miRNA-23a-3p targeted TGF-β2, ARG1, and miRNA-34a-5p (synonym: miRNA-449c based on IPA classification) targeted FoxP3 (Figure 5C). Also, we found miRNA-34a-5p to target GATA3 involved in Th2 differentiation and miRNA-30c-5p which was down-regulated to target SOCS1, a suppressor of cytokine signaling. These data suggested that AEA, by downregulating these miRNAs, may induce several of these anti-inflammatory molecules such as TGF- β2, ARG1, GATA3, and SOCS1.

### Validation of Micro-RNAs and Their Target Genes

**FIGURE 6:**
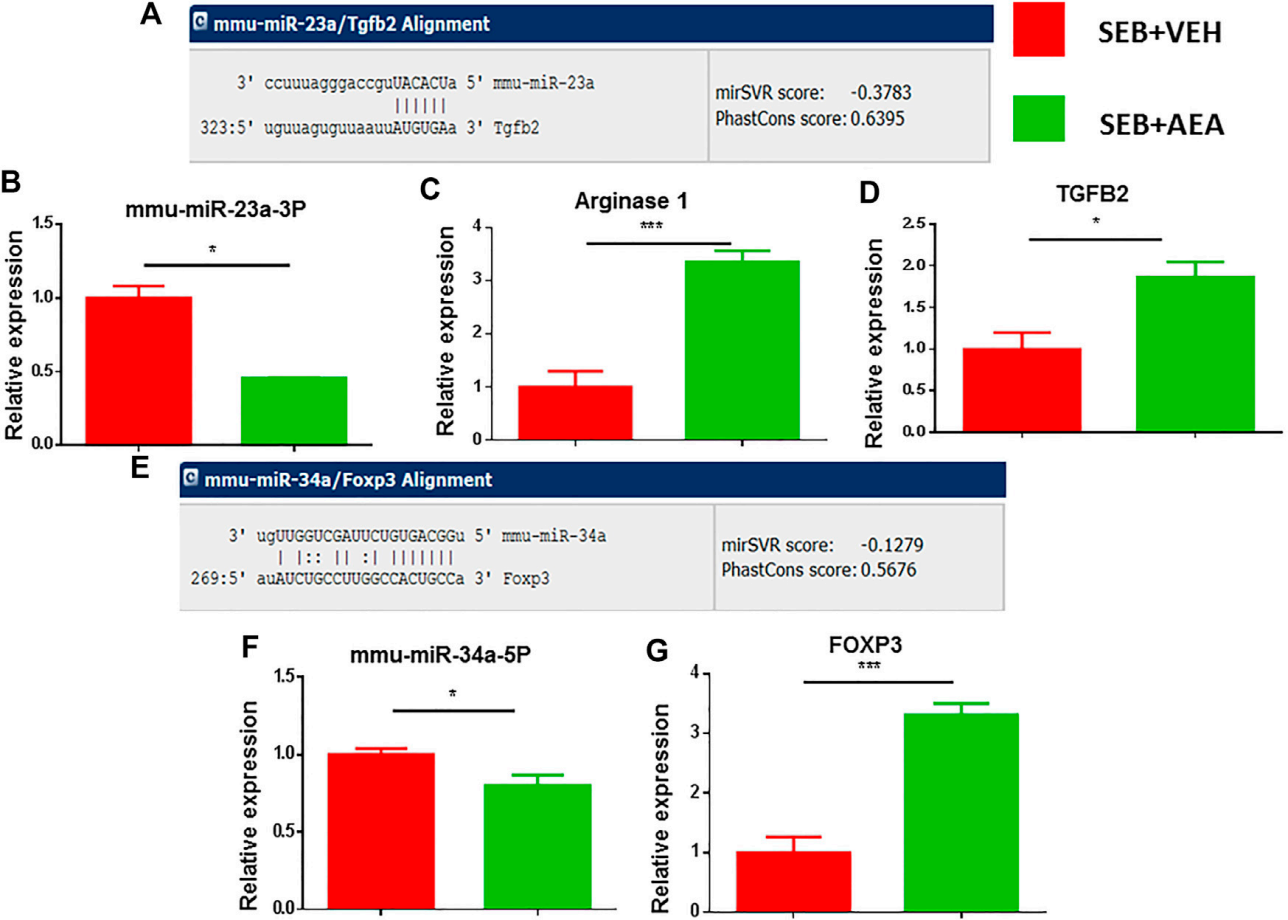
Validation of select miRNAs and targeted genes. Mice were treated with SEB and AEA as described in Figure 1 legend. Mononuclear cells from the lungs of both groups were isolated and screened for expression of miRNA expression with their targeted genes by qRT-PCR. **(A)** Binding affinity between miRNA 23a-3p and targeted genes including ARG1 and TGFβ2. **(B)** miRNA 23a-3p expression. **(C,D)** Expression of ARG1 and TGFβ2. **(E)** Binding affinity between miRNA 34a-5p and targeted gene FOXP3. **(F)** Expression of miRNA 34a-5p. **(G)** Expression FOXP3. Statistical significances as *p* ≤ 0. 05, **p ≤ 0. 01, ***p ≤ 0. 001.

We further confirmed miRNA-23a-3p and miRNA-34a-5p specific target genes using miRNA.org or TargetScan software. miRNA-23a-3p showed a strong binding affinity with its complementary 3′UTR region of TGF-β2 gene and miRNA-34-5pa showed a strong binding affinity with the 3′UTR region of the FoxP3 gene (Figures 6A,E). We then validated the expression of miRNA-23a-3p and miRNA-34a-5p and their respective genes (ARG1, TGF-β2, and FoxP3) by performing qRT-PCR using MNCs isolated from the lungs. Both miRNA-23a-3p and miRNA-34a-5p were significantly downregulated in SEB + AEA mice when compared to SEB + VEH counterparts (Figures 6B,F). Upon analysis of their respective target genes including ARG1, TGF-β2, and FoxP3 in the lung MNCs, there was a significant upregulation of both genes in cells from SEB + AEA mice when compared to SEB + VEH mice (Figures 6C,D,G).

### Analysis of Micro-RNA-23a-3p and Micro-RNA-34a-5p and Their Specific Target Gene Expression

**FIGURE 7:**
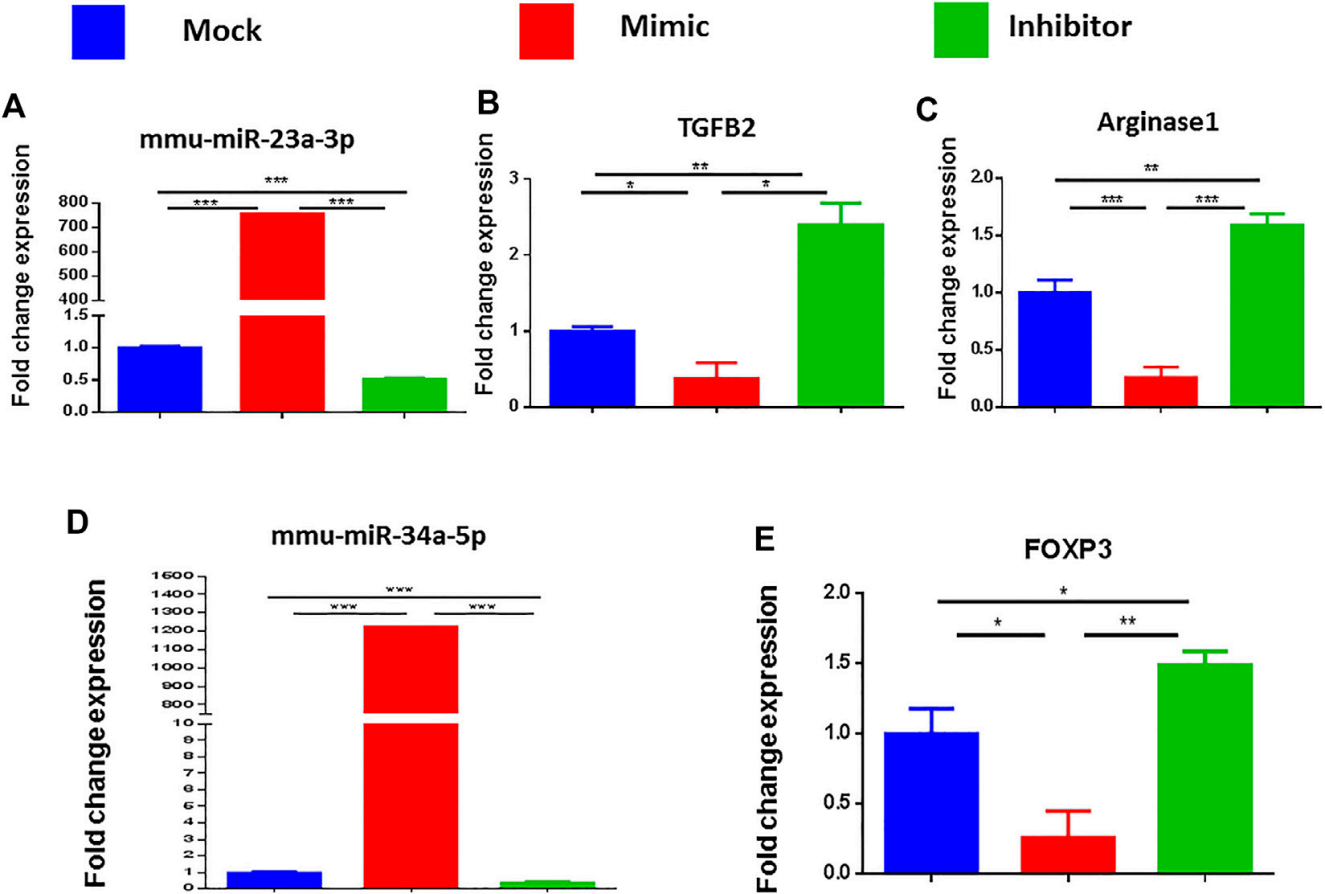
Validation of the genes targeted by miRNA 23a-3p and miRNA 34a-5p. Splenocytes of C57BL6 mice, cultured and activated with SEB overnight were transfected with mock, mimic and inhibitor of each of miRNA 23a-3p and miRNA 34a-5p. qRT-PCR was used to detect the levels of targeted genes**. (A)** Expression of miRNA 23a-3p. **(B)** Expression of TGFB2 gene. **(C)** Expression of arginase one gene **(D)** Expression of miRNA 34a-5p. **(E)** Expression of FOXP3. Statistical significances as *p* ≤ 0. 05, **p ≤ 0. 01, ***p ≤ 0. 001.

To corroborate that miRNA-23a-3p and miRNA-34a-5p specifically regulate the respective target genes (ARG1, TGF-β2, and FoxP3), we performed a series of transfection assays. To this end, SEB at a concentration of 1 μg/ml was used to activate splenocytes following overnight incubation. The following day, activated cells were then transfected with mock, mimic or inhibitor of miRNA-23a-3p, or miRNA-34a-5p. 48 h after transfection, we collected the cells for qRT-PCR. Based on the qRT-PCR analysis, we observed that there was a significant increase in miR-23a (Figure 7A) and miR-34a-5p expression (Figure 7D) in cells transfected with their respective mimics. In contrast, the cells that were transfected with respective inhibitors showed a significant decrease in miRNA-23a and miRNA-34a-5p expression. Upon analysis of their target genes, we noted that there was significant suppression of TGF-β2 and ARG1 in miR-23a mimic-transfected cells (Figures 7B,C) as well as FoxP3 expression in miRNA-34-5p mimic-transfected cells (Figure 7E), whereas there was complete reversal (increase) in their expression (Figures 7B,C,E) following inhibition of their respective miRNAs. These findings indicated a direct relationship with miRNA-23a-3p and miRNA-34a-5p expression with their target genes ARG1, TGF-β2, and FoxP3.

### Anandamide induces Myeloid-Derived Suppressor Cells and T Regulatory Cells

**FIGURE 8:**
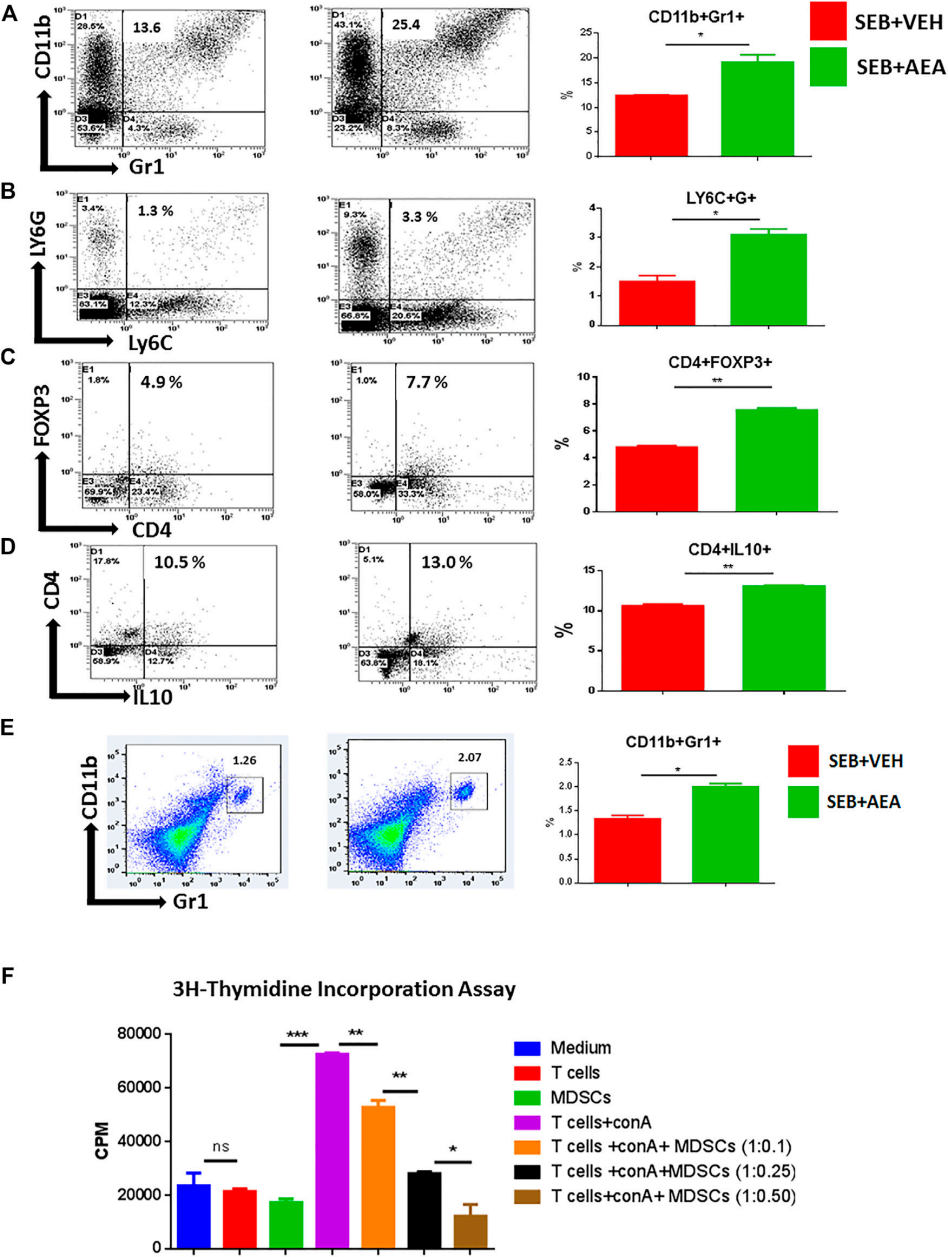
AEA induces MDSCs and Tregs in the lungs of SEB administered mice. Mice were treated with SEB and AEA as described in Figure 1 legend. The lung MNCs were next stained for markers to detect MDSCs and Tregs. Each panel shows a representative experiment depicting lung MNCs analyzed for various T cell markers. Data from five mice/group is presented in the form of vertical bars with Mean+/-SEM. **(A)** Cells double-stained for CD11b and Gr1. a representative experiment using flow cytometry. **(B)** Cells double-stained for LY6C and LY6G. **(C)** Cells double-stained for CD4 and FOXP3. **(D)** Cells double-stained for CD4 and IL10. **(E)** Cells from the spleens were double-stained for CD11b and Gr1. Vertical bars show data from five mice. **(F)** AEA-induced MDSCs were incubated with splenic T cells that were activated with ConA at different ratios to create different Tcell:MDSC ratios. T cell proliferation was assessed by ^3^H- Thymidine Incorporation Assay. Data from five mice/group is presented in the form of vertical bars with Mean+/-SEM. *p ≤ 0.05, **p ≤ 0.01, ***p ≤ 0.001.

The above studies indicated that AEA-regulated miRNAs might induce anti-inflammatory genes leading to the generation of suppressor cells such as MDSCs and T regulatory cells (Tregs). To test this, MNCs from the lungs of both SEB + AEA and SEB + VEH mice were assessed for the generation of MDSCs and their subsets (CD11b + Gr1+ and LY6C + LY6G+). Flow cytometry data showed that there was a significant increase in the percentage of MDSCs (CD11b + Gr1+) in SEB + AEA mice when compared to SEB + VEH mice in both the lungs and spleen (Figures 8A,E). Additionally, we noted that AEA caused increased percentages of both Ly6G^+^Ly6C^low^ granulocytic and Ly6G^−^Ly6C^high^ monocytic MDSCs (Figure 8B). Furthermore, upon analysis of Tregs, there was a significant increase in CD4 + FoxP3 + cells in SEB + AEA mice (Figure 8C) when compared to the SEB + VEH group. A significant increase of CD4 + IL10 + cells, a phenotype of regulatory Type 1 (Tr1)-like cells, was observed as well in SEB + AEA mice when compared to SEB + VEH mice, (Figure 8D). To confirm that the MDSCs induced by AEA were immunosuppressive, we performed an *in vitro* assay in which spleen T cells from naïve mice were activated with ConA in the presence of varying proportions of purified MDSCs. The Data shown in Figure 8F demonstrated that the MDSCs caused a dose-dependent decrease in T cell proliferation. These data together suggested that AEA was inducing immunosuppressive MDSCs and Tregs, consistent with the data shown in miRNA experiments.

## Discussion

In the present study, we explored the effect of AEA treatment in a SEB-induced mouse model of ARDS. Our results indicated that AEA indeed broadly improves clinical outcomes of lung function and inflammatory status following exposure to SEB. Mechanistically, AEA reduces the infiltration of proinflammatory T cell subsets and further suppresses splenic lymphocyte proliferation induced by SEB. Additional analyses suggested that such inflammatory control may be mediated, at least in part, *via* the suppression of two specific miRNAs, miRNA-23a-3p and miRNA-34a-5p, which directly regulate the expression of gene targets ARG1, TGF-β2, and FoxP3. As a result, the regulation of these miRNAs by AEA allows for an increase in immunosuppressive Tregs and MDSC populations.

ARDS is a life-threatening form of respiratory failure that affects heterogeneous populations. Currently, there is no FDA-approved drug to treat ARDS leading to high rates of mortality (Rao et al., 2015a). Treatment options are still limited because of which the mortality rate is almost 40% and according to United States healthcare data, the annual cost of treatment is more than $5 billion/year due to a long time of hospitalization and intensive medical care (Thornton Snider et al., 2012). With the advent of COVID-19, the additional healthcare burden will continue to rise (Bartsch et al., 2020) due to its ability to induce ARDS-like clinical features. ARDS is caused by a variety of insults which include pneumonia, sepsis, trauma, and certain viral infections. The common feature of ARDS also includes systemic heightened immune response leading to inflammation in the lungs, edema, alveolar damage, and respiratory failure. *Staphylococcus* Enterotoxin B (SEB) acts as a superantigen and activates a large proportion of T cells having certain Vβ T cell receptor-expressing cells (Vβ8) leading to cytokine storm and systemic inflammation (Nagarkatti et al., 2009; Rao et al., 2015a).

Cannabinoids have extensively been tested for their anti-inflammatory properties (Nagarkatti et al., 2009). Recently, we reported that Tetrahydrocannabinol (THC), the psychotropic compound found in cannabis that activates both CB1 and CB2, can suppress SEB-mediated ARDS (Mohammed et al., 2020a). However, whether endocannabinoids can suppress ARDS has not been tested previously. Such studies are important because the levels of endocannabinoids such as AEA can be regulated by inhibitors of Fatty Acid Amide Hydrolase (FAAH), an enzyme that breaks down AEA. AEA has a chemical structure that is different from THC, has lower affinity for CB_1_ receptors, and a much shorter duration of action both *in vitro* and *in vivo* (Justinova et al., 2005). Thus, while we found that 20 mg/kg of THC can block SEB-mediated ARDS when given even the same day as SEB (Mohammed et al., 2020a), with AEA, we found that it was necessary to give a higher dose of 40 mg/kg body weight, and also inject AEA prior to antigenic challenge (Jackson et al., 2014b). In this study, we used the endocannabinoid, AEA, as a mode of treatment for mice with ARDS due to the possibility that its levels can be increased using FAAH inhibitors. In fact, in a previous study, we noted that FAAH knockout mice or administration of FAAH inhibitor could upregulate endogenous AEA leading to suppression of autoimmune hepatitis (Hegde et al., 2008). The suppressive effect of AEA on TNFα and alveolar macrophages as well as on inflammatory cytokines IL6 and IL12 have been demonstrated in previous studies (Chiurchiù et al., 2015). AEA was also shown to activate CB1 receptors and cause a decrease in the production and release of IL-12 and IL-23 which induce inflammatory T cells namely Th1 and Th17 cells, respectively (Chiurchiù et al., 2016). In these studies, treatment of keratinocytes with AEA *in vitro* led to decreased induction of IFN-γ and IL-17 producing Th1 and Th17 cells respectively (Chiurchiù et al., 2016). The other key endocannabinoid, 2-AG, has also been shown to mediate anti-inflammatory properties. We have reported that naïve immune cells produced low levels of 2-AG but upon activation, they produced higher levels which in turn suppressed T cell proliferation (Sido et al., 2016). Also, 2-AG treatment (40 mg/kg) suppressed methylated Bovine Serum Albumin (BSA)-induced delayed-type hypersensitivity (DTH) response mediated by Th1 and Th17 cells (Sido et al., 2016). These studies suggested that immune cells upon activation may produce endocannabinoids which may act as negative regulators of the immune response (Sido et al., 2016).

In the current study, we noted that AEA induced significant levels of MDSCs. MDSCs are heterogeneous cells generated during inflammation and cancer development (Yang et al., 2020). Because of their remarkable ability to suppress T cell response (Bird, 2020), they are believed to promote tumor growth in cancer patients and suppress inflammation in autoimmune and inflammatory diseases (Elliott et al., 2018; De Cicco et al., 2020). Further, a previous study has shown that AEA induces MDSCs and cause immunosuppression during DTH response (Jackson et al., 2014a). There are two types of MDSCs: Ly6G^+^Ly6C^low^ granulocytic and Ly6G^−^Ly6C^high^ monocytic (Bird, 2020). We found that AEA increased the percentages of both these subsets but the granulocytic were induced to a greater extent when compared to the monocytic. There are many mechanisms through which MDSCs mediate the suppression of inflammation. Tumor-induced MDSC inhibit T cell proliferation by causing L-arginine depletion through arginase-1 activity (Ostrand-Rosenberg and Fenselau, 2018), and in the current study, we did find that AEA induced increased expression of arginase-1. MDSCs also produce immunosuppressive cytokines such as IL-10 and TGF-β (Eggert et al., 2020), and we found that AEA also induced increased expression of IL-10. The IL-10 produced by MDSCs has also been shown to induce Tregs (Park et al., 2018), which is consistent with the current study where we noted an increase in Tregs as well. It should be noted that both LPS and SEB have been used as a model to study lung inflammation. While LPS primarily induces neutrophils in the lungs, and SEB primarily induces mononuclear cells, SEB can also trigger, to a lesser extent, neutrophils when compared to LPS (Neumann et al., 1997). In the current study, we did not study the effect of AEA on neutrophils, which is a limitation.

In recent years, several studies including those from our lab have shown the critical role of miRNAs in gene expression (Singh et al., 2015; Alghetaa et al., 2018). We have shown that miRNAs are involved in the regulation of immune responses and in the upregulation of anti-inflammatory genes. However, there are limited studies on whether endocannabinoid-mediated attenuation of inflammation is associated with alterations in the miRNA of activated immune cells and there are no such studies in the regulation of inflammatory cells in the lungs seen during ARDS. In the current study, we, therefore, investigated the role of AEA in the regulation of miRNAs in lung infiltrating MNCs. We observed that treatment with AEA significantly altered the expression of several miRNAs in lung MNCs. AEA treatment led to upregulation of 59 miRNAs and downregulation of 77 miRNAs in comparison to VEH-treated counterparts. Using IPA analysis, we were able to narrow down our studies to miRNA-23a-3p which was downregulated and targeted ARG1 and TGFβ2 genes, while miRNA-34a-5p, also downregulated, targeted the FOXP3 gene. It is interesting to note that we found in a previous study that THC treatment was also able to decrease the expression of miRNA-34a-5p and induce FoxP3 (Mohammed et al., 2020a), similar to the action of AEA seen in the current study, thereby suggesting that miRNA-34a-5p is a critical miRNA that may be downregulated following treatment with cannabinoids such as THC and AEA. ARG1 encodes for arginase produced by the MDSCs which inhibits T cell proliferation by L-arginine depletion (Grzywa et al., 2020). These data were consistent with our observation that AEA also increased the proportions of MDSCs in the lung MNCs. FOXP3 is a transcription factor belonging to the forkhead/winged-helix family of transcriptional factors and is considered a master regulator of the development of Tregs (Ono, 2020). Moreover, it has been shown that FOXP3 expression is essential for Tregs development and function in mice (Fontenot et al., 2003). Additionally, TGFβ2 has been shown previously to drive Treg induction (Becker et al., 2018). In this study, we observed that treatment with AEA induced an increase in the percentages of FOXP3+ Tregs in the lungs of mice injected with SEB, consistent with the miRNA data. Importantly, we found using transfection studies involving mimic and inhibitor that miRNA-23a-3p targeted ARG1 and TGFβ2 expression, while miRNA-34a-5p targeted the FOXP3. The induction of Tregs and MDSCs by AEA is consistent with a previous observation that cannabinoids are potent inducers of these immunosuppressive cells. For example, both tetrahydrocannabinol (THC) and Cannabidiol (CBD) have been shown to induce MDSCs (Hegde et al., 2010; Hegde et al., 2011; Hegde et al., 2015; Sido et al., 2015a; Rao et al., 2015b; Elliott et al., 2018; Mohammed et al., 2020a). Also, AEA was shown to induce MDSCs even in naïve mice following i. p. injection through activation of mast cells to release MCP-1 (Jackson et al., 2014a). There are not many reports on the role of miRNA-23a on the regulation of inflammation. Interestingly, during septic insult, miRNA-23a expression was found to be decreased which was associated with increased autophagy and suppression of inflammatory mediators (Si et al., 2018). In this study, miRNA-23a was shown to target Autophagy related 12 (ATG12), which regulates autophagy (Si et al., 2018).

In summary, data from this study shows that AEA can effectively suppress SEB-induced ARDS in mice. Upon analysis of molecular mechanisms, we observed the role of miRNA-23a-3p in the regulation of TGF-β2 and ARG1 genes which in turn suppressed the inflammatory properties of SEB-induced MNCs in the lung. Similarly, miRNA-34a-5p directly regulates FOXP3 in lung infiltrating MNCs and the suppression of this miRNA by AEA might induce Tregs in the promotion of immune suppression following exposure to SEB.

## Data Availability

The datasets presented in this study can be found in online repositories. The names of the repository and accession numbers are as follows: https://www.ebi.ac.uk/arrayexpress/experiments/browse.html (EMBL-EBI, EMTAB-10274).

## References

[B1] Abohalaka R. Bozkurt T. E. Nemutlu E. Onder S. C. Sahin-Erdemli I. (2020). The effects of fatty acid amide hydrolase and monoacylglycerol lipase inhibitor treatments on lipopolysaccharide-induced airway inflammation in mice. Pulm. Pharmacol. Ther. 62, 101920. 10.1016/j.pupt.2020.101920 32416152

[B2] Al-Ghezi Z. Z. Miranda K. Nagarkatti M. Nagarkatti P. S. (2019). Combination of cannabinoids, delta9-tetrahydrocannabinol and cannabidiol, ameliorates experimental multiple sclerosis by suppressing neuroinflammation through regulation of miRNA-mediated signaling pathways. Front. Immunol. 10, 1921. 10.3389/fimmu.2019.01921 31497013 PMC6712515

[B3] Alghetaa H. Mohammed A. Sultan M. Busbee P. Murphy A. Chatterjee S. (2018). Resveratrol protects mice against SEB-induced acute lung injury and mortality by miR-193a modulation that targets TGF-β signalling. J. Cel. Mol. Med. 22, 2644–2655. 10.1111/jcmm.13542 PMC5908132 29512867

[B4] Ameri A. (1999). The effects of cannabinoids on the brain. Prog. Neurobiol. 58, 315–348. 10.1016/s0301-0082(98)00087-2 10368032

[B5] Barrie N. Manolios N. (2017). The endocannabinoid system in pain and inflammation: its relevance to rheumatic disease. Eur. J. Rheumatol. 4, 210–218. 10.5152/eurjrheum.2017.17025 29164003 PMC5685274

[B6] Bartsch S. M. Ferguson M. C. Mckinnell J. A. O’shea K. J. Wedlock P. T. Siegmund S. S. (2020). The potential health care costs and resource use associated with COVID-19 in the United States. Health Aff. 39, 927–935. 10.1377/hlthaff.2020.00426 PMC11027994 32324428

[B7] Becker W. Alrafas H. R. Wilson K. Miranda K. Culpepper C. Chatzistamou I. (2020). Activation of cannabinoid receptor 2 prevents colitis-associated colon cancer through myeloid cell de-activation upstream of IL-22 production. iScience 23, 101504. 10.1016/j.isci.2020.101504 32942172 PMC7501437

[B8] Becker W. Nagarkatti M. Nagarkatti P. S. (2018). miR-466a targeting of TGF-beta2 contributes to FoxP3(+) regulatory T cell differentiation in a murine model of allogeneic transplantation. Front. Immunol. 9, 688. 10.3389/fimmu.2018.00688 29686677 PMC5900016

[B9] Bird L. (2020). MDSC metabolite stuns T cells. Nat. Rev. Immunol. 20, 352–353. 10.1038/s41577-020-0336-z 32367052

[B10] Busbee P. B. Nagarkatti M. Nagarkatti P. S. (2015). Natural indoles, indole-3-carbinol (I3C) and 3,3’-diindolylmethane (DIM), attenuate staphylococcal enterotoxin B-mediated liver injury by downregulating miR-31 expression and promoting caspase-2-mediated apoptosis. PLoS One 10, e0118506. 10.1371/journal.pone.0118506 25706292 PMC4338211

[B11] Cannell I. G. Kong Y. W. Bushell M. (2008). How do microRNAs regulate gene expression? Biochem. Soc. Trans. 36, 1224–1231. 10.1042/bst0361224 19021530

[B12] Chiurchiù V. Battistini L. Maccarrone M. (2015). Endocannabinoid signalling in innate and adaptive immunity. Immunology 144, 352–364. 10.1111/imm.12441 25585882 PMC4557672

[B13] Chiurchiù V. Rapino C. Talamonti E. Leuti A. Lanuti M. Gueniche A. (2016). Anandamide suppresses proinflammatory T cell responses *in vitro* through type-1 cannabinoid receptor-mediated mTOR inhibition in human keratinocytes. J. Immunol. 197, 3545–3553. 10.4049/jimmunol.1500546 27694494

[B14] Costola-De-Souza C. Ribeiro A. Ferraz-De-Paula V. Calefi A. S. Aloia T. P. Gimenes-Junior J. A. (2013). Monoacylglycerol lipase (MAGL) inhibition attenuates acute lung injury in mice. PLoS One 8, e77706. 10.1371/journal.pone.0077706 24204926 PMC3808422

[B15] De Cicco P. Ercolano G. Ianaro A. (2020). The new era of cancer immunotherapy: targeting myeloid-derived suppressor cells to overcome immune evasion. Front. Immunol. 11, 1680. 10.3389/fimmu.2020.01680 32849585 PMC7406792

[B16] Devane W. Hanus L. Breuer A. Pertwee R. Stevenson L. Griffin G. (1992). Isolation and structure of a brain constituent that binds to the cannabinoid receptor. Science 258, 1946–1949. 10.1126/science.1470919 1470919

[B17] Di Marzo V. (2018). New approaches and challenges to targeting the endocannabinoid system. Nat. Rev. Drug Discov. 17, 623–639. 10.1038/nrd.2018.115 30116049

[B18] Diamond M. Peniston Feliciano H. L. Sanghavi D. Mahapatra S. (2020). Acute respiratory distress syndrome. Treasure Island, FL: StatPearls.

[B19] Dopkins N. Becker W. Miranda K. Walla M. Nagarkatti P. Nagarkatti M. (2020). Tryptamine attenuates experimental multiple sclerosis through activation of aryl hydrocarbon receptor. Front. Pharmacol. 11, 619265. 10.3389/fphar.2020.619265 33569008 PMC7868334

[B20] Eggert T. Dorn H. Sauter C. Schmid G. Danker-Hopfe H. (2020). RF-EMF exposure effects on sleep—age doesn’t matter in men! Environ. Res. 191, 110173. 10.1016/j.envres.2020.110173 32931791

[B21] Elliott D. M. Nagarkatti M. Nagarkatti P. S. (2016). 3,3’-Diindolylmethane ameliorates staphylococcal enterotoxin B-induced acute lung injury through alterations in the expression of microRNA that target apoptosis and cell-cycle arrest in activated T cells. J. Pharmacol. Exp. Ther. 357, 177–187. 10.1124/jpet.115.226563 26818958 PMC4809322

[B22] Elliott D. M. Singh N. Nagarkatti M. Nagarkatti P. S. (2018). Cannabidiol attenuates experimental autoimmune encephalomyelitis model of multiple sclerosis through induction of myeloid-derived suppressor cells. Front. Immunol. 9, 1782. 10.3389/fimmu.2018.01782 30123217 PMC6085417

[B23] Fan E. Brodie D. Slutsky A. S. (2018). Acute respiratory distress syndrome. JAMA 319, 698–710. 10.1001/jama.2017.21907 29466596

[B24] Fontenot J. D. Gavin M. A. Rudensky A. Y. (2003). Foxp3 programs the development and function of CD4+CD25+ regulatory T cells. Nat. Immunol. 4, 330–336. 10.1038/ni904 12612578

[B25] Grzywa T. M. Sosnowska A. Matryba P. Rydzynska Z. Jasinski M. Nowis D. (2020). Myeloid cell-derived arginase in cancer immune response. Front. Immunol. 11, 938. 10.3389/fimmu.2020.00938 32499785 PMC7242730

[B26] Hegde V. L. Hegde S. Cravatt B. F. Hofseth L. J. Nagarkatti M. Nagarkatti P. S. (2008). Attenuation of experimental autoimmune hepatitis by exogenous and endogenous cannabinoids: involvement of regulatory T cells. Mol. Pharmacol. 74, 20–33. 10.1124/mol.108.047035 18388242 PMC2828293

[B27] Hegde V. L. Nagarkatti M. Nagarkatti P. S. (2010). Cannabinoid receptor activation leads to massive mobilization of myeloid-derived suppressor cells with potent immunosuppressive properties. Eur. J. Immunol. 40, 3358–3371. 10.1002/eji.201040667 21110319 PMC3076065

[B28] Hegde V. L. Nagarkatti P. S. Nagarkatti M. (2011). Role of myeloid-derived suppressor cells in amelioration of experimental autoimmune hepatitis following activation of TRPV1 receptors by cannabidiol. PLoS One 6, e18281. 10.1371/journal.pone.0018281 21483776 PMC3069975

[B29] Hegde V. L. Singh U. P. Nagarkatti P. S. Nagarkatti M. (2015). Critical role of mast cells and peroxisome proliferator-activated receptor γ in the induction of myeloid-derived suppressor cells by marijuana cannabidiol *in vivo* . J. Immunol. 194, 5211–5222. 10.4049/jimmunol.1401844 25917103 PMC4433789

[B30] Jackson A. R. Hegde V. L. Nagarkatti P. S. Nagarkatti M. (2014a). Characterization of endocannabinoid-mediated induction of myeloid-derived suppressor cells involving mast cells and MCP-1. J. Leukoc. Biol. 95, 609–619. 10.1189/jlb.0613350 24319288 PMC3958741

[B31] Jackson A. R. Nagarkatti P. Nagarkatti M. (2014b). Anandamide attenuates Th-17 cell-mediated delayed-type hypersensitivity response by triggering IL-10 production and consequent microRNA induction. PLoS One 9, e93954. 10.1371/journal.pone.0093954 24699635 PMC3974854

[B32] Justinova Z. Solinas M. Tanda G. Redhi G. H. Goldberg S. R. (2005). The endogenous cannabinoid anandamide and its synthetic analog R(+)-methanandamide are intravenously self-administered by squirrel monkeys. J. Neurosci. 25, 5645–5650. 10.1523/jneurosci.0951-05.2005 15944392 PMC2562767

[B33] Krol J. Loedige I. Filipowicz W. (2010). The widespread regulation of microRNA biogenesis, function and decay. Nat. Rev. Genet. 11, 597–610. 10.1038/nrg2843 20661255

[B34] Li X. Ma X. (2020). Acute respiratory failure in COVID-19: is it “typical” ARDS? Crit. Care 24, 198. 10.1186/s13054-020-02911-9 32375845 PMC7202792

[B35] Macfarlane L. A. Murphy P. R. (2010). MicroRNA: biogenesis, function and role in cancer. Curr. Genomics. 11, 537–561. 10.2174/138920210793175895 21532838 PMC3048316

[B36] Matthay M. A. Zimmerman G. A. (2005). Acute lung injury and the acute respiratory distress syndrome. Am. J. Respir. Cel. Mol. Biol 33, 319–327. 10.1165/rcmb.f305 PMC2715340 16172252

[B37] Mechoulam R. Ben-Shabat S. Hanus L. Ligumsky M. Kaminski N. E. Schatz A. R. (1995). Identification of an endogenous 2-monoglyceride, present in canine gut, that binds to cannabinoid receptors. Biochem. Pharmacol. 50, 83–90. 10.1016/0006-2952(95)00109-d 7605349

[B38] Mohammed A. Alghetaa H. K. Zhou J. Chatterjee S. Nagarkatti P. Nagarkatti M. (2020b). Protective effects of Delta(9)-tetrahydrocannabinol against enterotoxin-induced acute respiratory distress syndrome are mediated by modulation of microbiota. Br. J. Pharmacol. 177 (22), 5078–5095. 10.1111/bph.15226 32754917 PMC7436585

[B39] Mohammed A. Alghetaa H. Sultan M. Singh N. P. Nagarkatti P. Nagarkatti M. (2020a). Administration of delta9-tetrahydrocannabinol (THC) post-staphylococcal enterotoxin B exposure protects mice from acute respiratory distress syndrome and toxicity. Front. Pharmacol. 11, 893. 10.3389/fphar.2020.00893 32612530 PMC7308536

[B40] Mohammed A. Hasan F. K. A. Miranda K. Wilson K. N P. S. Cai G. (2020c). Delta9-Tetrahydrocannabinol prevents mortality from acute respiratory distress syndrome through the induction of apoptosis in immune cells, leading to cytokine storm suppression. Int. J. Mol. Sci. 21, 6244. 10.3390/ijms21176244 PMC7503745 32872332

[B41] Muller C. Morales P. Reggio P. H. (2018). Cannabinoid ligands targeting TRP channels. Front. Mol. Neurosci. 11, 487. 10.3389/fnmol.2018.00487 30697147 PMC6340993

[B42] Nagarkatti P. Pandey R. Rieder S. A. Hegde V. L. Nagarkatti M. (2009). Cannabinoids as novel anti-inflammatory drugs. Future Med. Chem. 1, 1333–1349. 10.4155/fmc.09.93 20191092 PMC2828614

[B43] Neumann B. Engelhardt B. Wagner H. Holzmann B. (1997). Induction of acute inflammatory lung injury by staphylococcal enterotoxin B. J. Immunol. 158, 1862–1871. 9029127

[B44] O’neill L. A. Sheedy F. J. Mccoy C. E. (2011). MicroRNAs: the fine-tuners of Toll-like receptor signalling. Nat. Rev. Immunol. 11, 163–175. 10.1038/nri2957 21331081

[B45] Ono M. (2020). Control of regulatory T‐cell differentiation and function by T‐cell receptor signalling and Foxp3 transcription factor complexes. Immunology 160, 24–37. 10.1111/imm.13178 32022254 PMC7160660

[B46] Ostrand-Rosenberg S. Fenselau C. (2018). Myeloid-derived suppressor cells: immune-suppressive cells that impair antitumor immunity and are sculpted by their environment. J. Immunol. 200, 422–431. 10.4049/jimmunol.1701019 29311384 PMC5765878

[B47] Pandey R. Mousawy K. Nagarkatti M. Nagarkatti P. (2009). Endocannabinoids and immune regulation. Pharmacol. Res. 60, 85–92. 10.1016/j.phrs.2009.03.019 19428268 PMC3044336

[B48] Park M. J. Lee S. H. Kim E. K. Lee E. J. Baek J. A. Park S. H. (2018). Interleukin-10 produced by myeloid-derived suppressor cells is critical for the induction of tregs and attenuation of rheumatoid inflammation in mice. Sci. Rep. 8, 3753. 10.1038/s41598-018-21856-2 29491381 PMC5830490

[B49] Pertwee A. A. Waterhouse P. M. (2005). Plant and animal microRNAs: similarities and differences. Funct. Integr. Genomics 5, 129–135. 10.1007/s10142-005-0145-2 15875226

[B50] Pinchuk I. V. Beswick E. J. Reyes V. E. (2010). Staphylococcal enterotoxins. Toxins 2, 2177–2197. 10.3390/toxins2082177 22069679 PMC3153290

[B51] Rao R. Nagarkatti P. Nagarkatti M. (2015a). Role of miRNA in the regulation of inflammatory genes in staphylococcal enterotoxin B-induced acute inflammatory lung injury and mortality. Toxicol. Sci. 144, 284–297. 10.1093/toxsci/kfu315 25564423 PMC4372662

[B52] Rao R. Nagarkatti P. S. Nagarkatti M. (2015b). Δ9Tetrahydrocannabinol attenuates Staphylococcal enterotoxin B-induced inflammatory lung injury and prevents mortality in mice by modulation of miR-17-92 cluster and induction of T-regulatory cells. Br. J. Pharmacol. 172, 1792–1806. 10.1111/bph.13026 25425209 PMC4376457

[B53] Rieder S. A. Nagarkatti P. Nagarkatti M. (2011). CD1d-independent activation of invariant natural killer T cells by staphylococcal enterotoxin B through major histocompatibility complex class II/T cell receptor interaction results in acute lung injury. Infect. Immun. 79, 3141–3148. 10.1128/iai.00177-11 21628519 PMC3147567

[B54] Rieder S. A. Nagarkatti P. Nagarkatti M. (2012). Multiple anti-inflammatory pathways triggered by resveratrol lead to amelioration of staphylococcal enterotoxin B-induced lung injury. Br. J. Pharmacol. 167, 1244–1258. 10.1111/j.1476-5381.2012.02063.x 22646800 PMC3504991

[B55] Shamran H. Singh N. P. Zumbrun E. E. Murphy A. Taub D. D. Mishra M. K. (2017). Fatty acid amide hydrolase (FAAH) blockade ameliorates experimental colitis by altering microRNA expression and suppressing inflammation. Brain Behav. Immun. 59, 10–20. 10.1016/j.bbi.2016.06.008 27327245 PMC5154806

[B56] Si X. Cao D. Chen J. Nie Y. Jiang Z. Chen M. Y. (2018). miR‑23a downregulation modulates the inflammatory response by targeting ATG12‑mediated autophagy. Mol. Med. Rep. 18, 1524–1530. 10.3892/mmr.2018.9081 29845275 PMC6072189

[B57] Sido J. M. Nagarkatti P. S. Nagarkatti M. (2016). Production of endocannabinoids by activated T cells and B cells modulates inflammation associated with delayed-type hypersensitivity. Eur. J. Immunol. 46, 1472–1479. 10.1002/eji.201546181 27064137 PMC5206973

[B58] Sido J. M. Nagarkatti P. S. Nagarkatti M. (2015b). Role of endocannabinoid activation of peripheral CB1 receptors in the regulation of autoimmune disease. Int. Rev. Immunol. 34, 403–414. 10.3109/08830185.2014.921165 24911431 PMC4261058

[B59] Sido J. M. Nagarkatti P. S. Nagarkatti M. (2015a). Δ9-Tetrahydrocannabinol attenuates allogeneic host-versus-graft response and delays skin graft rejection through activation of cannabinoid receptor 1 and induction of myeloid-derived suppressor cells. J. Leukoc. Biol. 98, 435–447. 10.1189/jlb.3a0115-030rr 26034207 PMC4541500

[B60] Singh N. P. Abbas I. K. Menard M. Singh U. P. Zhang J. Nagarkatti P. (2015). Exposure to diethylstilbestrol during pregnancy modulates microRNA expression profile in mothers and fetuses reflecting oncogenic and immunological changes. Mol. Pharmacol. 87, 842–854. 10.1124/mol.114.096743 25753120 PMC4407731

[B61] Thornton Snider J. Romley J. A. Wong K. S. Zhang J. Eber M. Goldman D. P. (2012). The Disability burden of COPD. COPD: J. Chronic Obstructive Pulm. Dis. 9, 513–521. 10.3109/15412555.2012.696159 22721264

[B62] Tomar S. E. Zumbrun E. Nagarkatti M. Nagarkatti P. S. (2015). Protective role of cannabinoid receptor 2 activation in galactosamine/lipopolysaccharide-induced acute liver failure through regulation of macrophage polarization and microRNAs. J. Pharmacol. Exp. Ther. 353, 369–379. 10.1124/jpet.114.220368 25749929 PMC4407720

[B63] Van Egmond N. Straub V. M. Van Der Stelt M. (2021). Targeting endocannabinoid signaling: FAAH and MAG lipase inhibitors. Annu. Rev. Pharmacol. Toxicol. 61, 441–463. 10.1146/annurev-pharmtox-030220-112741 32867595

[B64] Walker J. M. Huang S. M. (2002). Endocannabinoids in pain modulation. Prostaglandins, Leukot. Essent. Fatty Acids 66, 235–242. 10.1054/plef.2001.0361 12052039

[B65] Wu K. Xiu Y. Zhou P. Qiu Y. Li Y. (2019). A new use for an old drug: carmofur attenuates lipopolysaccharide (LPS)-Induced acute lung injury *via* inhibition of FAAH and NAAA activities. Front. Pharmacol. 10, 818. 10.3389/fphar.2019.00818 31379583 PMC6659393

[B66] Yang Z. Guo J. Weng L. Tang W. Jin S. Ma W. (2020). Myeloid-derived suppressor cells-new and exciting players in lung cancer. J. Hematol. Oncol. 13, 10. 10.1186/s13045-020-0843-1 32005273 PMC6995114

[B67] Zhao Y. Ridge K. Zhao J. (2017). Acute lung injury, repair, and remodeling: pulmonary endothelial and epithelial biology. Mediators Inflamm. 2017, 9081521. 10.1155/2017/9081521 28392632 PMC5368364

